# Exploring the Microbiome and Functional Metabolism of Fermented Camel Milk (Shubat) Using Metagenomics

**DOI:** 10.3390/foods14071102

**Published:** 2025-03-22

**Authors:** Sagyman Zhadyra, Fei Tao, Ping Xu

**Affiliations:** 1 State Key Laboratory of Microbial Metabolism, School of Life Sciences and Biotechnology, Shanghai Jiao Tong University, Shanghai 200240, China; sagyman@alumni.sjtu.edu.cn (S.Z.); pingxu@sjtu.edu.cn (P.X.); 2 Laboratory of Biotechnology, Research Institute for Biotechnology and Ecology, Zhetysu University, Taldykorgan 040009, Kazakhstan

**Keywords:** Shubat, metagenomics, functional metabolism, fermented camel milk, microbial diversity, functional annotation

## Abstract

Shubat is a traditional fermented camel milk drink that originated in Central Asia, with especially deep cultural roots in Kazakhstan. However, systematic studies on the microbial ecology and functional genes of Shubat remain scarce. As a distinctive fer-mented food, its microbial diversity and functional properties have not been fully ex-plored. This study investigates the microbial diversity and functional potential of Shubat by using advanced metagenomic techniques. Its microbial community is mainly composed of bacteria (96.6%), with Lactobacillus, Lactococcus, and Streptococcus being the dominant genera. Functional annotations through EggNOG, KEGG, and CAZy databases highlighted the metabolic versatility of Shubat’s microbiota. Key pathways included amino acid and carbohydrate metabolism, vitamin biosynthesis, and central carbon metabolism, emphasizing their roles in fermentation and nutritional enhancement. The identification of various enzymes related to chemical synthesis further emphasizes the contribution of the microbiota to Shubat’s unique flavor and texture. This study not only provides an important basis for the scientific understanding of Shubat but also expands the application possibilities of fermented food in the field of health and nutrition and confers modern value and significance to traditional food. This integration of science and tradition has not only facilitated the development of food microbiology but also paved new pathways for the global dissemination of traditional foods and the development of functional foods.

## 1. Introduction

Shubat, a traditional fermented camel milk beverage, is deeply rooted in Kazakh culture and is valued for its nutritional benefits, particularly in regions where it is widely consumed. Its production involves a natural fermentation process that transforms camel milk into a beverage with enhanced nutritional and therapeutic properties [1]. The unique composition of camel milk, including higher levels of unsaturated fatty acids, bioactive proteins, and vitamins compared to bovine milk, provides an ideal medium for microbial growth and activity, making Shubat a promising model for studying microbe-driven fermentation [2,3].

Fermentation not only extends the shelf life of dairy products but also enriches their flavor, texture, and functional properties. The microbial communities involved in this process play a crucial role in shaping the final product through complex biochemical transformations, such as carbohydrate fermentation, vitamin synthesis, and the production of bioactive compounds [4,5]. These metabolic activities contribute to the nutritional and health-promoting qualities of fermented dairy products, including their antimicrobial, antioxidant, and immunomodulatory effects [6,7].

Naturally fermented foods contain unique microbial communities, including bacteria, archaea, fungi, and viruses, which influence the characteristics of the final product during fermentation [8,9,10]. Many of these microorganisms have been proven to be important components of fermented milk and play a crucial role in the fermentation process. However, due to their widespread, unculturable nature, researchers were unable to conduct detailed sequencing and analyses until just a few years ago. High-throughput sequencing and bioinformatics have revolutionized our understanding of microbial communities in fermented foods. Metagenomics enables the comprehensive profiling of microbial diversity and functional potential [11]. By annotating protein-coding genes and mapping metabolic pathways, researchers can uncover the roles of microbial taxa in fermentation processes and their contributions to the nutritional and sensory attributes of the final product [12,13]. Databases such as EggNOG, KEGG, and CAZy provide valuable resources for identifying functional enzymes, metabolic pathways, and carbohydrate-active enzymes, facilitating a deeper understanding of the functional microbiome [14,15].

This study aims to investigate the microbial community structure and functional potential of Shubat through metagenomic analysis. By leveraging advanced annotation tools and databases, we seek to elucidate the metabolic pathways and enzymatic activities that underpin Shubat’s fermentation process and contribute to its nutritional and therapeutic properties. Understanding these microbial dynamics not only enhances our knowledge of traditional fermented foods but also offers practical insights for optimizing fermentation practices and developing novel functional dairy products tailored for health and wellness.

## 2. Materials and Methods

### 2.1. Sample Collection

Seven Shubat samples were collected from local families in four regions of Kazakhstan: Almaty (S1, S2), Kyzylorda (S3), Zhetisu (S4, S7), and Turkestan (S5, S6). Each sample is a composite of three subsamples, which were collected from different households within the same village. Although some samples originated from the same region, they represent independent samples from distinct villages located considerable distances apart. Variations in fermentation practices, hygiene conditions, and environmental factors among households may contribute to differences in the microbial composition of Shubat.

Following collection, the subsamples were thoroughly homogenized, and 50 mL of each composite sample was transferred into sterilized tubes using sterile pipettes. The samples were pre-chilled, transported in insulated containers with ice packs, and stored at −80 °C until further analysis.

### 2.2. Total DNA Extraction

Chemical Lysis: A total of 790 μL of sterile lysis buffer (4 M guanidine thiocyanate, 10% N-lauroyl sarcosine, 5% N-lauroyl sarcosine-0.1 M phosphate buffer, and pH 8.0) were added to 2 mL centrifuge tubes containing 1× *g* of glass beads (0.1 mm, BioSpec Products, Inc., Bartlesville, OK, USA) and Shubat samples. After vortexing, the mixture was incubated at 70 °C for 1 h.

Physical Disruption: After incubation, the mixture was bead-beaten for 10 min using a tissue grinder at maximum speed and centrifuged at 12,000 rpm for 10 min. The supernatant (800 µL) was transferred to new tubes.

Nucleic Acid Purification: DNA was extracted using the E.Z.N.A.^®^ Stool DNA Kit (Omega Bio-tek, Inc., Norcross, GA, USA) following the manufacturer’s protocol, including steps for protein and RNA removal. DNA quality and quantity were assessed using NanoDrop 2000 spectrophotometry (Thermo Fisher Scientific, Waltham, MA, USA), 1% agarose gel electrophoresis, and Qubit 3.0 fluorometry. DNA samples were stored at −20 °C for further analysis.

### 2.3. Library Construction and Illumina Sequencing

DNA (0.5–1 µg) was fragmented (~350 bp) using Covaris and processed with the Nextera XT DNA Library Preparation Kit (Illumina, San Diego, CA, USA). The library preparation involved end-repair, adapter ligation, PCR amplification, and bead purification. Libraries were quantified and pooled for s equencing. Sequencing was conducted on the Illumina NovaSeq 6000 platform (Illumina, San Diego, CA, USA), employing bridge amplification to generate clusters and sequencing by synthesis to acquire paired-end reads.

### 2.4. Data Quality Control and Assembly

Raw sequencing reads were filtered to remove low-quality reads, adapters, and short sequences (<75 bp). Reads with >10% ambiguous bases or terminal quality scores < 20 were excluded. Clean reads were assembled using MEGAHIT (v1.2.9) with default parameters. Contigs were assessed for quality, including N50 and total bases.

### 2.5. Bioinformatics Analysis

Taxonomic Annotation: Gene sequences were annotated using BLAST (v2.13.0) against the NR database (e-value < 1 × 10^−5^). Abundance profiles at various taxonomic levels (domain to species) were constructed based on gene abundance. Functional Annotation: Gene functions were annotated using KEGG, eggNOG, and CAZy databases. Functional abundances were calculated using KO, Pathway, EC, and Module categories.

### 2.6. Statistical Analysis

All statistical analyses were performed using Python (v3.12.3). Alpha diversity indices, including Shannon, Simpson, and Pielou’s Evenness, were calculated using the scikit-bio package (v0.6.3) to evaluate microbial richness and evenness across samples. The Kruskal–Wallis test was used to assess significant differences in alpha diversity between sample groups.

Beta diversity was assessed using the Bray–Curtis dissimilarity index, and Principal Coordinate Analysis (PCoA) was conducted to visualize sample clustering patterns. The statistical significance of group differences was tested using PERMANOVA (Permutational Multivariate Analysis of Variance) with 999 permutations. A *p*-value of <0.05 was considered statistically significant.

## 3. Results

### 3.1. Species Annotation Results

**Figure 1:**
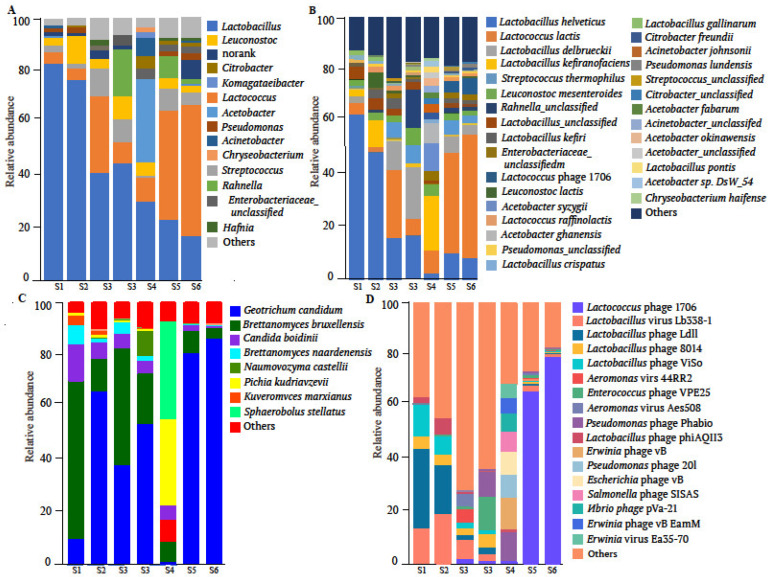
Relative abundance of the microbial community of Shubat samples at the genus (**A**) and species level (**B**) (abundance > 1%). Bar diagram of fungal (**C**) and viral (**D**) community structure in Shubat samples (abundance > 5%).

The microbial community structure of Shubat samples was analyzed at the domain, genus, and species levels. At the domain level, bacteria dominated the community, accounting for approximately 96.6% of total reads (260,089,516 reads), followed by viruses (3.18%), eukaryotes (0.34%), and archaea (0.22%, ~6000 reads). At the genus level, five genera represented over 80% of the microbial community: *Lactobacillus* (16.22–82.76%), *Lactococcus* (4.45–50.43%), *Streptococcus* (5.01–10.47%), *Leuconostoc* (2.54–10.6%), and *Acetobacter* (0.11–31.71%). Eight other genera, including *Rahnella*, *Pseudomonas*, and *Citrobacter*, were identified as subdominant groups, each accounting for 1–4% of the total abundance (Figure 1A).

At the species level, 5062 species were detected, of which 30 had an abundance of 1% or higher. The six most abundant species—*Lactobacillus helveticus*, *Lactococcus lactis*, *Lactobacillus delbrueckii*, *Lactobacillus kefiranofaciens*, *Streptococcus thermophilus*, and *Leuconostoc mesenteroides*—constituted over 60% of the total community. Among these, *Lb. helveticus* was the most dominant species, with a relative abundance ranging from 2.7% to 63.63% across samples (Figure 1B). The high prevalence of lactic acid bacteria (LAB) highlights their pivotal role in the fermentation process of Shubat, contributing to lactic acid production and flavor development.

Eukaryotic sequences, although less abundant (0.34% of total reads), were dominated by the genus *Geotrichum*, particularly *Geotrichum. candidum* (74.11% of fungal sequences), which may contribute to the aroma and texture of Shubat. A total of 160 eukaryotic microbial taxa were identified. Among these, the order *Saccharomycetes* accounted for 96.17% of all eukaryotic fungi. Based on the community structure profiles across seven samples, eight dominant fungal species with relative abundances exceeding 5% were identified: *G. candidum*, *Candida bruxellensis*, *Candida boidinii*, *Brettanomyces naardenensis*, *Naumovozyma castellii*, *Pichia kudriavzevii*, and *Sphaerobolus stellatus* (Figure 1C).

In addition to bacteria and fungi, viral sequences were detected, with 801 species identified, primarily belonging to the families Siphoviridae (73.93%) and Myoviridae (14.47%). At the species level, five dominant viral species accounted for more than 80% of the detected viral sequences: *Lactococcus* phages (51.04%), *Lactobacillus* phages (19.66%), *Aeromonas* phages (6.38%), *Enterococcus* phages (3.23%), and *Streptococcus* phages (2.83%). Among the Lc. phages, the most abundant species was *Lactococcus* phage 1706. The abundance of this phage varied across the seven samples, with the highest proportions observed in S15 (48.17%) and S16 (50.95%) (Figure 1D).

### 3.2. Microbial Community Diversity Analysis

#### 3.2.1. Alpha Diversity Analysis

The microbial diversity among samples was evaluated using Shannon, Simpson, and Pielou’s Evenness indices (Table S1). S5 exhibited the highest Shannon diversity (3.42), while S1 had the lowest (2.08), indicating that S5 harbored the most diverse microbial community. Similarly, the Simpson index showed that S1 had the lowest evenness (Simpson = 0.59), suggesting that a few species dominated this sample, whereas S5 had the most evenly distributed microbial community (Simpson = 0.92).

Kruskal–Wallis tests showed no statistically significant differences in alpha diversity among sample groups (*p* > 0.05), indicating that while microbial diversity varied numerically, the differences were not statistically significant.

#### 3.2.2. Beta Diversity Analysis

The compositional differences among microbial communities were assessed using Bray–Curtis dissimilarity and visualized with Principal Coordinates Analysis (PCoA) (Figure S1). The first two principal coordinates (PC1 and PC2) explained 44.47% and 31.14% of the total variation, respectively.

The PCoA plot revealed that samples S1–S3 clustered together, whereas S5–S7 formed a separate group, suggesting compositional differences between these two groups. However, PERMANOVA analysis did not detect statistically significant differences among groups (pseudo-F = 1.54, *p* = 0.181), indicating that the observed clustering patterns were not strongly structured by sample groups (Table S2).

### 3.3. Functional Annotation and Analysis

Functional annotation of the Shubat metagenome using the EggNOG, KEGG, and CAZy databases revealed a wide range of metabolic potential. Among the predicted protein-coding genes, 38.65–47.88% were classified into COG functional categories. These annotations demonstrated that the dominant functions in Shubat samples were associated with metabolism, supported by significant contributions from pathways related to information storage and cellular processes.

#### 3.3.1. COG Functional Classification Analysis

**Figure 2:**
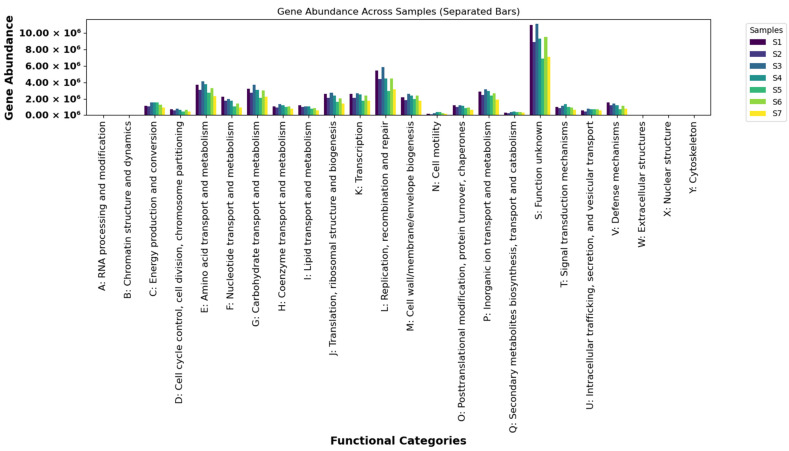
Clusters of orthologous groups (COG) functional classification (the *x*-axis represents the 24 different groups; the *y*-axis refers to the frequency of genes).

The EggNOG-based COG annotation assigned the identified genes to 24 functional categories. Most of the genes were enriched in metabolism-related pathways, including amino acid transport and metabolism, carbohydrate transport and metabolism, and energy production and conversion. High-abundance categories such as lipid metabolism, coenzyme transport, and secondary metabolite biosynthesis were prominent, highlighting the metabolic versatility of Shubat microbial communities. Low-abundance categories (e.g., A, B, and W) exhibited minimal gene presence, indicating less diversity in these functions. Overall, the dominance of metabolism-related genes aligns with the physiological requirements for microbial growth and the fermentation process (Figure 2).

#### 3.3.2. KEGG Pathway Analysis

**Figure 3:**
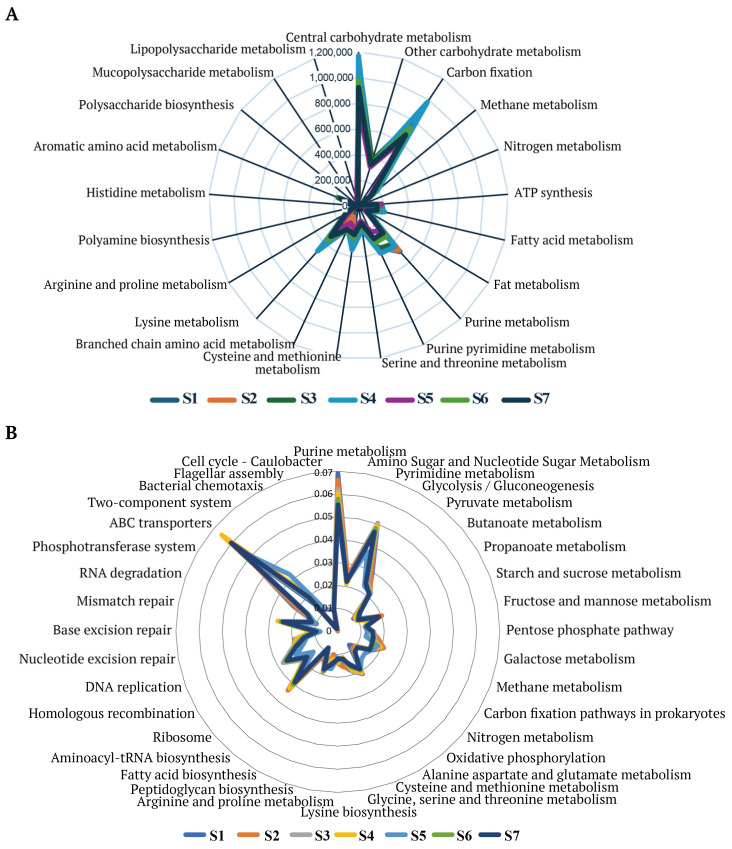
Radar map of the enriched KEGG pathways (**A**). Radar diagram of gene enrichment analysis in KEGG module (**B**).

Using the KEGG database, a total of 312,649 genomic sequences were aligned, which were annotated to 288 metabolic pathways, 1754 enzymes, 461 modules, and 6034 KO entries. The gene abundance of KEGG pathways in the Shubat metagenome is shown in Figure 3A.

Nutritional and metabolic features: At the KEGG secondary pathway level, several key pathways related to the metabolism of proteins, carbohydrates, and lipids were identified. Carbohydrate metabolism pathways included glycolysis/gluconeogenesis, pyruvate metabolism, butanoate metabolism, propanoate metabolism, starch and sucrose metabolism, fructose and mannose metabolism, amino sugar and nucleotide sugar metabolism, pentose phosphate pathway, and galactose metabolism. Amino acid metabolism pathways covered the alanine, aspartate, and glutamate metabolism; the biosynthesis of valine, leucine, and isoleucine; the cysteine and methionine metabolism; the glycine, serine, and threonine metabolism; the lysine biosynthesis; and arginine and proline metabolism. Lipid metabolism pathways included fatty acid biosynthesis and related processes. These findings highlight the essential roles of microbial communities in nutrient metabolism and functional enhancement during the fermentation of Shubat.

Vitamin biosynthesis potential: Several metabolic pathways associated with folate, riboflavin, and pantothenic acid biosynthesis were detected, highlighting the potential for Shubat to contribute to dietary benefits (Table 1). The relative abundance of these pathways across Shubat samples is expressed as genes that count per million reads, reflecting the number of annotated genes linked to each metabolic function within the metagenomic dataset. Higher values suggest greater genetic potential for vitamin biosynthesis within the microbial community.

Specifically, Table 1 highlights the high abundance of key vitamin biosynthesis modules, including the folate biosynthesis module (M00126) and the riboflavin biosynthesis module (M00125), both of which were consistently enriched across different samples. Additionally, ascorbate (vitamin C) biosynthesis modules, including pathways for animals (M00129) and plants (M00114), were identified, although these modules appear to have limited direct association with the Shubat microbial community. The detection of cobalamin (M00122) and biotin (M00123) biosynthesis modules, albeit at lower abundance, further suggests that the Shubat microbiota may have some capacity for synthesizing these essential vitamins. Table 1 shows the abundance of some vitamin biosynthesis modules.

In addition, KEGG module analysis revealed the dominance of central carbon metabolism (CCM), which is fundamental to microbial life (Figure 3B). The CCM includes glycolysis, the pentose phosphate pathway (PPP), and the TCA cycle, which are essential for energy generation and carbon flux. Other enriched pathways included nucleotide metabolism, secondary metabolite biosynthesis, and amino acid biosynthesis, further reflecting the functional adaptability of the Shubat microbial community.

#### 3.3.3. Carbohydrate-Active Enzyme (CAZy) Analysis

**Figure 4:**
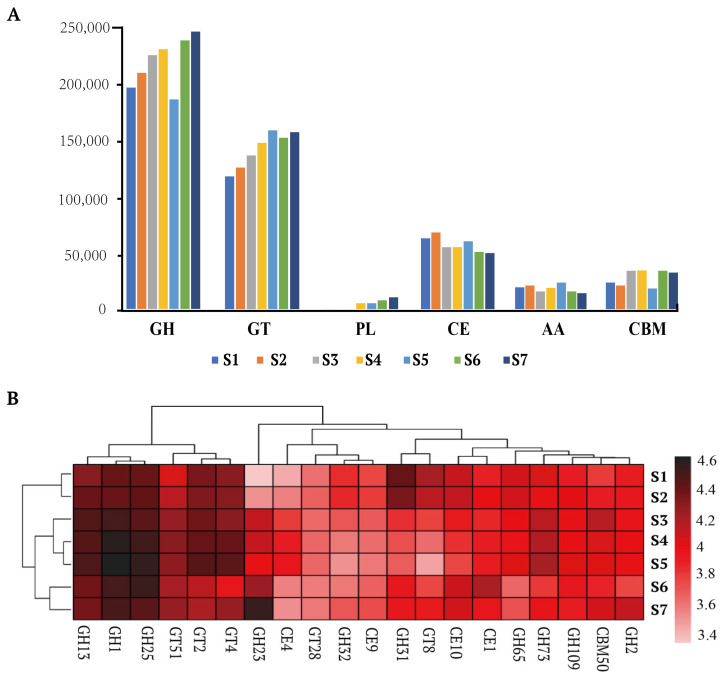
Number of genes encoding different carbohydrate-active enzymes (CAZy) classes of CAZy database (**A**). The top twenty families of CAZy are involved in carbohydrate metabolism in Shubat samples (**B**).

Annotation of carbohydrate-active enzymes (CAZymes) revealed 214 enzyme families categorized into six functional classes: GHs, GTs, PLs, CEs, AAs, and CBMs. Glycoside hydrolases (GHs) and glycosyltransferases (GTs) were the most abundant classes, indicating strong polysaccharide degradation and glycosylation activities. These enzymes play a crucial role in the breakdown and modification of complex carbohydrates, contributing to the flavor and texture of fermented milk products (Figure 4A).

As shown in Figure 4B, annotation through the CAZy database identified the top 20 families potentially involved in carbohydrate metabolism in Shubat. These include Glycoside Hydrolases (GHs): GH23, GH31, GH73, GH109, GH2, GH65, GH32, GH13, GH1, GH25. Glycosyl Transferases (GTs): GT51, GT8, GT28, GT4, GT2. Carbohydrate Esterases (CEs): CE1, CE10, CE4, CE9. Carbohydrate-Binding Modules (CBMs): CBM50. The CAZy analysis revealed that among the glycoside hydrolase families, GH13, GH1, and GH25 contributed the most significantly.

## 4. Discussion

### 4.1. Microbial Community Structure Analysis

Metagenomic sequencing provides a comprehensive and culture-independent approach to studying microbial communities, offering significant advantages over traditional culture-based microbiological methods [16]. Unlike conventional techniques, which rely on the ability to isolate and cultivate viable microorganisms, metagenomics enables the direct identification and functional characterization of entire microbial consortia, including unculturable and low-abundance taxa [11]. This is particularly valuable in analyzing fermented dairy products such as Shubat, where microbial interactions and metabolic activities play a crucial role in product quality and potential health benefits.

Previous studies utilizing culture-dependent methods have primarily identified dominant LAB species, such as *Lactobacillus* spp. and *Lactococcus* spp., in fermented camel milk [8,9,17]. However, the metagenomic analysis in this study provided a broader perspective, revealing additional taxa, including low-abundance but functionally relevant microorganisms such as *G. candidum* and bacteriophages, which were previously undetectable through culture-based techniques. Comparatively, metagenomic data also provided deeper insights than traditional 16S rRNA sequencing, capturing a more diverse bacterial community and identifying genera such as *Komagataeibacter* and *Chryseobacterium*, which were not prominently detected in earlier studies [9,18]. This comprehensive approach enriches our understanding of the Shubat microbiome, highlighting the contribution of previously overlooked taxa and their functional roles in carbohydrate metabolism, vitamin biosynthesis, and antimicrobial compound production. These findings further support the classification of Shubat as a functional food, given its potential to harbor beneficial microbial interactions that contribute to both fermentation quality and possible health benefits.

To statistically assess variations in alpha diversity, Kruskal–Wallis tests were performed on Shannon, Simpson, and Evenness indices, revealing no significant differences among sample groups (*p* > 0.05). This suggests that although microbial diversity varied numerically, it was not significantly influenced by geographical origin or specific fermentation practices. However, beta diversity analysis provided additional insights into community-level compositional changes, as certain sample groups showed distinct clustering patterns in the PCoA plot. While these differences were not statistically significant, the observed grouping trends indicate that environmental or processing factors may still shape the Shubat microbiome.

#### 4.1.1. Bacterial Diversity of Shubat

A metagenomic analysis of Shubat’s bacterial composition revealed a highly diverse microbial ecosystem, aligning with recent studies on fermented camel milk. The bacterial composition at the genus level highlighted that *Lactobacillus* and *Lactococcus* were the dominant genera, representing 16.22–82.76% and 4.45–50.43% relative abundances, respectively. The balance between these genera likely shifts dynamically over the fermentation process, consistent with the findings of Bao et al., who demonstrated that *Lactococcus* decreases over time while *Lactobacillus* increases, suggesting their complementary roles in the development of Shubat’s organoleptic and functional properties [8].

At the species level, Shubat was characterized by a core group of LAB, including *Lb. helveticus*, *Lc. lactis*, and *Leu. mesenteroides*. These species are known for their probiotic potential, contributing to the flavor, texture, and potential health benefits of fermented dairy products [19,20,21]. The prominence of *Lb. helveticus* in this study (2.7–63.63% abundance) is noteworthy and aligns with previous research that identified LAB as a key component of fermented camel milk [18,22].

Interestingly, the detection of additional species, such as *Lb. kefiranofaciens* and *Lc. raffinolactis* underscores the diverse microbial ecology of Shubat, which may be influenced by regional variations in raw milk composition and fermentation practices [23,24]. The presence of minor genera, such as *Rahnella* and *Pseudomonas*, further highlights the broad biodiversity within this ecosystem, although their functional roles require further investigation [25].

#### 4.1.2. Dominant Fungal Species in Shubat

Fungal communities, although minor in abundance, play a pivotal role in shaping the sensory and functional characteristics of Shubat. A comprehensive analysis of Shubat’s fungal diversity identified 160 eukaryotic microbial species, with the class Saccharomycetes accounting for 96.17% of all fungal sequences. This dominance of yeasts aligns with their historical role in fermenting dairy and alcoholic beverages, including kefir and mare’s milk-derived drinks such as Koumiss [26,27].

Among the identified fungi, *G. candidum* was the most prevalent species. Known for its acid tolerance, *G. candidum* contributes to the flavor and aroma profiles of cheeses and fermented milk products, as extensively documented in dairy science [28]. The second most abundant species was *Brettanomyces bruxellensis,* a yeast often associated with the spontaneous fermentation of beer and other alcoholic beverages. Its role in fermented dairy products such as Shubat is less understood, although Baubekova et al. also detected this species in Shubat and Koumiss, suggesting its contribution to flavor complexity [9,17].

Prior studies using PCR-DGGE methods, such as those by Rahman et al. and Akhmetsadykova et al., revealed yeasts such as *Kluyveromyces marxianus*, *Saccharomyces unisporus*, and *Candida ethanolica*, alongside unidentified *Geotrichum* species, as core members of Shubat’s fungal microbiome [29,30]. While these studies highlighted key yeast species, they lacked the depth afforded by next-generation sequencing (NGS) employed in this research. Here, we not only confirmed previously identified species but also identified additional members, such as *Naumovozyma castellii* and *Pichia kudriavzevii*, broadening the understanding of Shubat’s fungal diversity.

The functional contributions of these fungi, particularly *G. candidum* and *B. bruxellensis*, underscore their potential roles in Shubat’s fermentation and flavor development. The dominance of *G. candidum*, known for its activity in traditional dairy fermentations, highlights its significance in Shubat’s natural fermentation process. Meanwhile, the presence of *B. bruxellensis*, although uncommon in fermented milk, may represent an adaptation unique to the Shubat ecosystem, contributing to its distinct sensory attributes.

#### 4.1.3. Viral Composition in Shubat

This study identified 801 viral species across seven Shubat samples. The most dominant viral species was *Lc.* phage 1706, contributing 23.17% of the total viral abundance. Its distribution was notably higher in samples S15 (48.17%) and S16 (50.95%). Originally isolated in 1995 from a failed cheese production batch in France, *Lc*. phage 1706 is a virulent phage known for its impact on dairy fermentations [31]. Given that *Lc. lactis* is a key starter culture in global dairy production, the presence of virulent phages such as *Lc.* phage 1706 has been linked to fermentation failures and reduced cheese quality [32]. Consequently, phage contamination is a critical concern in controlled dairy production.

In traditional fermented dairy products such as Shubat, LAB and phages coexist in the same environment over extended periods. This long-term coexistence has likely driven the evolution of robust phage defense mechanisms in LAB strains, such as CRISPR systems and restriction–modification systems [33]. These mechanisms might explain why LAB hosts maintain high abundance despite the presence of virulent phages. For instance, the dominant LAB species identified in this study (e.g., *Lc. lactis* and *Lb. helveticus*) coexist with their respective phages, highlighting the dynamic interplay between phages and bacteria in Shubat’s microbial ecosystem.

The diversity and dominance of LAB-targeting phages in Shubat emphasize their potential roles in shaping microbial community structure and influencing fermentation outcomes. While phages are typically viewed as detrimental in controlled fermentations, in traditional systems such as Shubat, they may contribute to the development of unique microbial equilibria, enhancing the flavor and functional properties of the final product.

### 4.2. Functional Annotation Analysis

The functional annotation of the Shubat metagenome provides insightful information regarding the metabolic potential and microbial diversity of the Shubat fermentation process. This study employed multiple databases—EggNOG, KEGG, and CAZy—to examine the functional categories and pathways associated with Shubat microbial communities, revealing significant metabolic versatility and potential health benefits.

#### 4.2.1. COG Functional Classification Analysis

The COG analysis identified a strong dominance of metabolism-related functions within the Shubat microbiome, accounting for 38.65–47.88% of the annotated genes. These findings are consistent with similar metagenomic studies in fermented products, where microbial communities play a key role in metabolic processes essential for fermentation [34,35]. Notably, pathways related to amino acid metabolism, carbohydrate metabolism, and energy production were predominant. Such metabolic pathways are central to microbial growth and fermentation, emphasizing their importance in the fermentation process of Shubat, which likely influences its nutritional profile and taste.

The prominence of lipid metabolism, coenzyme transport, and secondary metabolite biosynthesis categories highlights the versatility of Shubat’s microbial community in supporting a variety of biochemical processes [36]. Similar results were observed in the microbiomes of other dairy products, such as kefir and yogurt, where the diversity of metabolic pathways contributes to the complex flavors and health-promoting properties of the final product [37,38,39]. However, the low-abundance categories (e.g., categories A, B, and W) suggest that some functions are less active in Shubat, which may reflect specific microbial taxa with specialized functions or limitations in their functional diversity.

#### 4.2.2. KEGG Pathway Analysis

KEGG pathway analysis identified key pathways involved in amino acid metabolism, such as lysine and valine biosynthesis, and carbohydrate metabolism, including starch and sucrose metabolism, suggesting that Shubat’s microbial community contributes significantly to the nutritional profile of the product. This is in line with studies that have reported the role of fermentation in enhancing the bioavailability of nutrients, including amino acids and carbohydrates [40]. Additionally, the detection of pathways associated with vitamin biosynthesis, including folate, riboflavin, and pantothenic acid, aligns with previous findings that fermented foods can serve as sources of bioactive compounds beneficial for human health [41].

The identification of vitamin C and cobalamin biosynthesis modules is of particular interest, as it suggests the potential for Shubat to provide these essential nutrients. While the presence of ascorbate biosynthesis pathways in Shubat’s microbial community is less pronounced, detecting cobalamin and biotin biosynthesis at lower abundance may still offer functional benefits [42,43].

The dominance of central carbon metabolism (CCM), including glycolysis, the pentose phosphate pathway (PPP), and the TCA cycle, further emphasizes the metabolic activity of the Shubat microbiota. CCM is fundamental to microbial energy production and carbon flux, and its dominance in the Shubat metagenome supports the idea that these pathways are critical to the success of the fermentation process and the metabolic adaptability of the microbial community [44].

#### 4.2.3. CAZy Analysis

The CAZy analysis revealed a high abundance of carbohydrate-active enzymes (CAZymes), particularly GHs and GTs, which are responsible for polysaccharide degradation and glycosylation activities. The presence of these enzymes reflects the ability of Shubat’s microbial community to degrade complex carbohydrates, such as lactose and other polysaccharides present in camel milk. This enzymatic activity is crucial to producing bioactive compounds and developing flavor and texture in fermented dairy products [45,46].

GHs play a critical role in breaking down complex sugars into simpler sugars, which are essential for microbial fermentation. In Shubat, the prevalence of GHs indicates that these enzymes contribute significantly to the fermentation process, potentially influencing both the nutritional value and sensory properties of the product [47]. Similarly, GTs modify carbohydrates, affecting the texture and flavor of fermented products. The identification of these enzymes in Shubat suggests that the microbial community is well-equipped to modify and enhance the taste and mouthfeel of the final product, providing additional consumer appeal [48].

Similarly to the findings from many metagenomic projects, the GH13 family was identified as the most dominant glycoside hydrolase family [49,50,51]. This dominance may be attributed to the fact that GH13 is the largest sequence-based GH family, encompassing a variety of enzyme activities and substrate specificities for α-glycosidic bonds [52]. The GH13 family is commonly regarded as the primary α-amylase family, acting on substrates containing α-glycosidic bonds [53]. It primarily includes enzymes such as α-glucosidase (EC 3.2.1.10 and EC 3.2.1.20), α-amylase (EC 3.2.1.1), glucan glucosidase (EC 3.2.1.70), branching enzyme (EC 2.4.1.18), and 4-α-glucanotransferase (EC 2.4.1.25). The glycoside hydrolases of the GH25 family are lysozymes (EC 3.2.1.17). These lysozymes may inhibit the growth of Staphylococcus aureus and other pathogenic microorganisms in Shubat [54].

## 5. Conclusions

This study provides a comprehensive metagenomic characterization of Shubat, a traditional fermented camel milk product, shedding light on its diverse microbial community and functional potential. The dominance of bacteria, particularly LAB such as *Lb. helveticus* and *Lc. Lactis*, underscores their pivotal role in the fermentation process, contributing to lactic acid production, flavor development, and the overall quality of Shubat. Additionally, the detection of fungal taxa such as *G. candidum* and the identification of phages associated with key bacterial genera reflect the complexity and balance of microbial interactions within the Shubat microbiota.

Functional annotations using EggNOG, KEGG, and CAZy databases highlighted the metabolic versatility of Shubat’s microbial community. Genes associated with central carbon metabolism, amino acid biosynthesis, carbohydrate metabolism, and vitamin biosynthesis were abundant, emphasizing the nutritional and health-promoting attributes of Shubat. The presence of pathways linked to folate, riboflavin, and pantothenic acid biosynthesis further suggests that Shubat may serve as a functional food with potential dietary benefits.

However, while metagenomic analysis provides valuable insights into the genetic potential of the microbial community, the mere presence of functional genes does not directly confirm enzymatic activity or its impact on sensorial quality properties. Further validation through transcriptomic and metabolomic (metabolite profiling) approaches is necessary to establish a definitive link between genetic potential and actual biochemical activity. Future studies should focus on isolating and characterizing key microbial strains, assessing their enzymatic functions, and evaluating their contributions to the flavor, texture, and bioactive properties of Shubat. These efforts could enhance industrial production, optimize fermentation conditions, and further support the global appreciation of this unique traditional beverage.

## Figures and Tables

**Table 1:** Relative abundance of vitamin biosynthesis modules in Shubat samples (expressed as gene counts per million reads).

Modules	S1	S2	S3	S4	S5	S6	S7	Definition
M00126	87,186	87,941	79,462	84,788	65,367	63,582	56,931	Folate biosynthesis (genes per million reads)
M00114	40,426	41,904	41,105	40,314	42,411	36,005	33,733	Ascorbic acid biosynthesis (plant pathway)
M00125	12,642	25,768	33,743	36,125	38,846	31,054	31,515	Riboflavin biosynthesis
M00119	17,479	24,192	29,167	29,532	21,888	27,604	25,812	Pantothenic acid biosynthesis
M00129	29,475	28,593	24,405	27,100	16,887	20,047	17,807	Ascorbic acid biosynthesis (animal pathway)
M00124	14,037	14,364	15,664	19,006	15,394	15,746	15,714	Pyridoxal biosynthesis
M00122	1831	10,572	13,428	5385	11,303	7824	6933	Cobalamin (vitamin B12) biosynthesis
M00123	711	1147	2818	6537	11,655	3079	3000	Biotin biosynthesis

## Data Availability

The original contributions presented in the study are included in the article/Supplementary Material, further inquiries can be directed to the corresponding author.

## Supplementary Materials

The following supporting information can be downloaded at: https://www.mdpi.com/article/10.3390/foods14071102/s1, Figure S1. Principal Coordinates Analysis (PCoA) based on Bray-Curtis dissimilarity of microbial communities in Shubat samples; Table S1: Alpha diversity indices (Shannon, Simpson, and Evenness) for different samples; Table S2: Results of PERMANOVA analysis based on Bray-Curtis dissimilarity, showing pseudo-F values, p-values, permutations, and R² effect sizes for microbial community composition differences.
