# Supplementary material for: Exploring the Microbiome and Functional Metabolism of Fermented Camel Milk (Shubat) Using Metagenomics
Source: Foods. 2025 Mar 22;14(7):1102. doi: 10.3390/foods14071102 (PMC11989172; doi:10.3390/foods14071102)
Supplement: Supplementary file 1 [file foods-14-01102-s001.zip › foods-3495814-supplementary.pdf]

Supplementary materials

Figure S1. Principal Coordinates Analysis (PCoA) based on Bray-Curtis dissimilarity of microbial communities in Shubat samples.

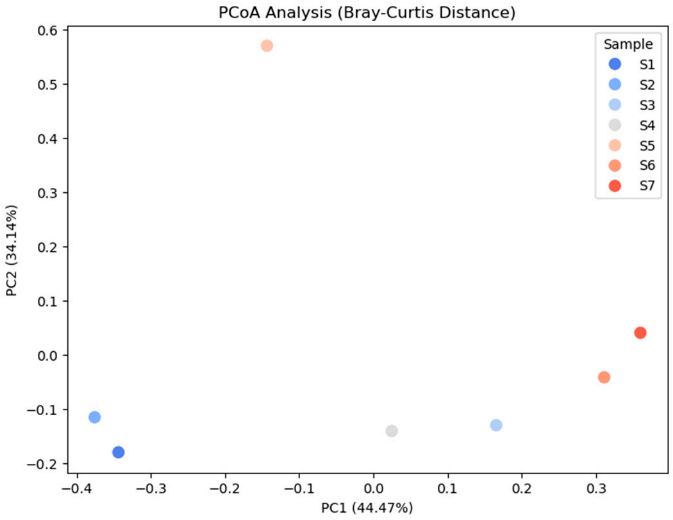

Table S1: Alpha diversity indices (Shannon, Simpson, and Evenness) for different samples.

Sample	Shannon	Simpson	Evenness
S1	2.08	0.59	0.27
S2	2.50	0.74	0.32
S3	3.32	0.89	0.41
S4	3.17	0.89	0.39
S5	3.42	0.92	0.46
S6	3.12	0.83	0.38
S7	2.81	0.76	0.34

Table S2: Results of PERMANOVA analysis based on Bray-Curtis dissimilarity, showing pseudo-F values, p-values, permutations, and R^2 effect sizes for microbial community composition differences.

Comparison	pseudo-F	p-value	Permutations	R^2 (Effect Size)
All samples	1.54	0.181	999	0.12
